# Pitfalls Associated with Discriminating Mixed-Species Biofilms by Flow Cytometry

**DOI:** 10.3390/antibiotics9110741

**Published:** 2020-10-27

**Authors:** Tânia Grainha, Andreia P. Magalhães, Luís D. R. Melo, Maria O. Pereira

**Affiliations:** Centre of Biological Engineering, LIBRO—Laboratório de Investigação em Biofilmes Rosário Oliveira, University of Minho, Campus de Gualtar, 4710-057 Braga, Portugal; taniagrainha@ceb.uminho.pt (T.G.); amagalhaes@ceb.uminho.pt (A.P.M.)

**Keywords:** *Pseudomonas aeruginosa*, *Candida albicans*, mixed-species biofilm analysis, flow cytometry

## Abstract

Since biofilms are ubiquitous in different settings and act as sources of disease for humans, reliable methods to characterize and quantify these microbial communities are required. Numerous techniques have been employed, but most of them are unidirectional, labor intensive and time consuming. Although flow cytometry (FCM) can be a reliable choice to quickly provide a multiparametric analysis, there are still few applications on biofilms, and even less on the study of inter-kingdom communities. This work aimed to give insights into the application of FCM in order to more comprehensively analyze mixed-species biofilms, formed by different *Pseudomonas aeruginosa* and *Candida albicans* strains, before and after exposure to antimicrobials. For comparison purposes, biofilm culturability was also assessed determining colony-forming units. The results showed that some aspects, namely the microbial strain used, the morphological state of the cells and the biofilm matrix, make the accurate analysis of FCM data difficult. These aspects were even more challenging when double-species biofilms were being inspected, as they could engender data misinterpretations. The outcomes draw our attention towards the need to always take into consideration the characteristics of the biofilm samples to be analyzed through FCM, and undoubtedly link to the need for optimization of the processes tailored for each particular case study.

## 1. Introduction

The ability of microorganisms to form a biofilm is an important feature in clinical, industrial and environmental settings [1]. Biofilms are well-structured microbial communities adhered to biotic or abiotic surfaces enclosed within a self-produced extracellular polymeric matrix. The matrix is the major structural component of the biofilm [2] and is denominated as an extracellular polymeric substance (EPS). The EPS is composed of polysaccharides, proteins, nucleic acids, lipids and other biopolymers such as humic substances [3]. Most natural biofilms are polymicrobial and all members of the community contribute with their own EPS components, resulting in a more complex matrix [4].

The biofilms associated with numerous infectious diseases (e.g., infections of oral cavity, otitis, cystic fibrosis) are described as being of a polymicrobial nature with bacteria coexisting with pathogenic yeasts or filamentous fungi [5]. *Pseudomonas aeruginosa* is a ubiquitous bacterium and an opportunistic pathogen frequently isolated from healthy humans as part of the human microbiota and can coexist in mixed infections with the polymorphic fungus *Candida albicans* [6,7]. *C. albicans* is a commensal yeast able to initiate invasive growth and develop health problems in compromised individuals [8,9]. Notably, *C. albicans* is one of the few fungal species causing disease in humans [10]. *P. aeruginosa* and *C. albicans* represent an example of co-infection and are commonly related with chronic and healthcare-associated infections [11,12,13]. From a clinical point of view, it is crucial to entirely characterize the biofilm populations (single- and mixed-species) and understand the mechanisms underlying the changes that occur during co-infection as result of the established interactions. The monitoring of polymicrobial communities is routinely performed by culture-dependent approaches that require appropriate selective media and optimal growth conditions and is hindered by the presence of cells in a viable but poorly culturable state or underestimated by the presence of cellular aggregates [14,15]. Under these circumstances, standard microbiology methods are often unsuitable to diagnose polymicrobial biofilm infections. Molecular methodologies, such as quantitative real-time PCR (qPCR) [16,17] or fluorescence in situ hybridization using peptide nucleic acids (PNA FISH) [18,19,20], have helped in the characterization of members of microbial communities, affording a specific and sensitive quantification of specific species in biofilm samples that are unable to be detected by culturable methods. Nevertheless, these approaches cannot distinguish cellular subpopulations. Thus, flow cytometry (FCM) could present an accurate alternative to the analysis of biofilm cells, since it enables detailed investigation of cellular subpopulations due to its ability to perform multiparametric single-cell analysis [21]. The analysis of biofilm communities by this technique is particularly useful, since it allows a quick achievement of cell counts and provides an overview about the type of cells present in the samples, namely their size and complexity. In addition, this method allows for the discrimination between live and dead cells, also providing information about damaged cells [22]. Based on this, the goal of this study was to explore FCM analysis to characterize and discriminate mixed-species biofilm samples before and after being challenged by antimicrobial treatments.

Accordingly, the aspects that can influence FCM analysis as well as tips to circumvent them are presented through this work.

## 2. Results

### 2.1. Planktonic versus Biofilm Cells

**Figure 1:**
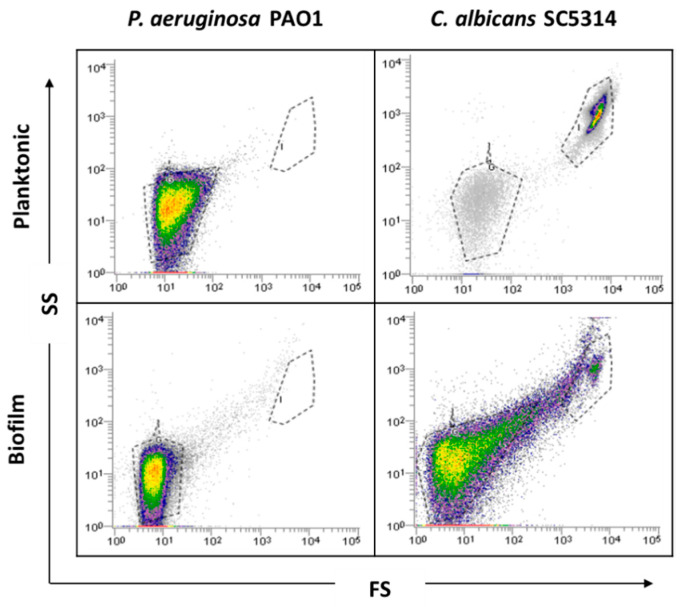
Representative dot plots obtained for planktonic cells and single biofilms of *P. aeruginosa* PAO1 and *C. albicans* SC5314 by flow cytometry (FCM).

Before biofilm analysis by FCM, an optimization of the process is desirable to adjust all sample analysis parameters. Firstly, as bacteria might be in the FCM detection limit, owing to its small size, a preliminary optimization was performed in order to guarantee the accurate detection of *P. aeruginosa* and thus the reliability of the results. Due to the complexity and heterogeneity of biofilms, optimizations using planktonic cells were performed. Since *P. aeruginosa* and *C. albicans* have distinct sizes and the flow cytometer allows for separation by size and complexity, it was assumed that their distinction on mixed communities should be well achieved using the same dyes. As can be observed in the dot plot (Figure 1), it was possible to define gates for each single-species planktonic culture tested.

**Figure 2:**
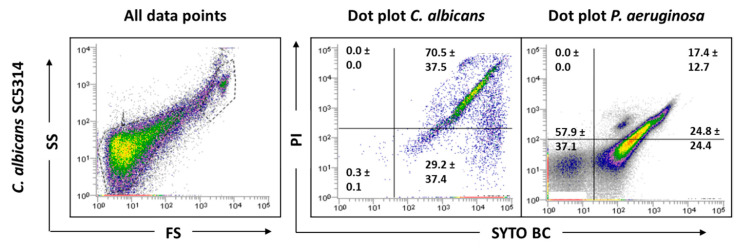
Representative dot plots obtained for *C. albicans* SC5314 biofilm by FCM. In ‘all data points’, the dot plots SS (Side Scatter) × FS (Forward Scatter) are represented, as acquired in the logarithm. Dot plots of *P. aeruginosa* and *C. albicans* represent the areas delineated to represent bacteria and fungi, respectively.

Before examining the mixed-species biofilms, single-species biofilms of both species were also analyzed. The results obtained for these biological samples showed that cells from *C. albicans* biofilm populations are very different on cell size and complexity from the planktonic cells, leading to gating adjustments (Figure 1). In addition to the typical fungal cell gate previously defined for the planktonic cultures, several fluorescently labeled events up to the demarcated bacterial region were detected (Figure 2). To check whether this feature was particular for the *C. albicans* SC5314 strain, two more strains of each studied species (*P. aeruginosa* and *C. albicans*) were analyzed both on planktonic and biofilm states. Although this effect was not observed for *P. aeruginosa* PAO1, two more *P. aeruginosa* strains were also analyzed to strengthen the absence of that behavior. While this effect was not observed for any of the *P. aeruginosa* strains (Supplementary Figure S1), it was consistent on all *C. albicans* strains tested (Supplementary Figure S2).

### 2.2. Effect of Hyphae and Biofilm Matrix on Flow Cytometry Analysis

In an attempt to understand the factors generating the differences observed in the *C. albicans* biofilm populations in comparison with planktonic cultures, two possibilities were inspected, the impact of hyphal growth and the effect of the biofilm matrix. Since *C. albicans* biofilm cells present different phases of growth, it is expected that cells with distinct hyphal lengths are also present. Additionally, the biofilms were grown in Roswell Park Memorial Institute (RPMI) 1640, a medium that favors hyphal growth; therefore, more elongated hyphae could appear in these samples compared with planktonic cultures [23]. Based on this knowledge, the possibility of hyphal growth introducing some heterogeneity in the population, with a consequent interference in the results, was raised. To evaluate this hypothesis, planktonic cultures of *C. albicans* SC5314 grown in Sabouraud Dextrose Broth (SDB) and in RPMI supplemented with serum 2% (*v/v*), to induce hyphal cell growth, were analyzed. It was noticed that the fungal population which had hyphae induction became more heterogenous (Supplementary Figure S3), thus allowing us to verify that the presence of hyphae could account for the observed differences. However, a closer inspection of the results for *C. albicans* 547096, a strain that does not have the ability to form hyphae in both planktonic and biofilm conditions, allowed us to notice a change between both modes of growth (Supplementary Figure S2). Despite it being clear that hyphal growth has some influence on the results, this did not entirely explain what happened in biofilm samples.

**Figure 3:**
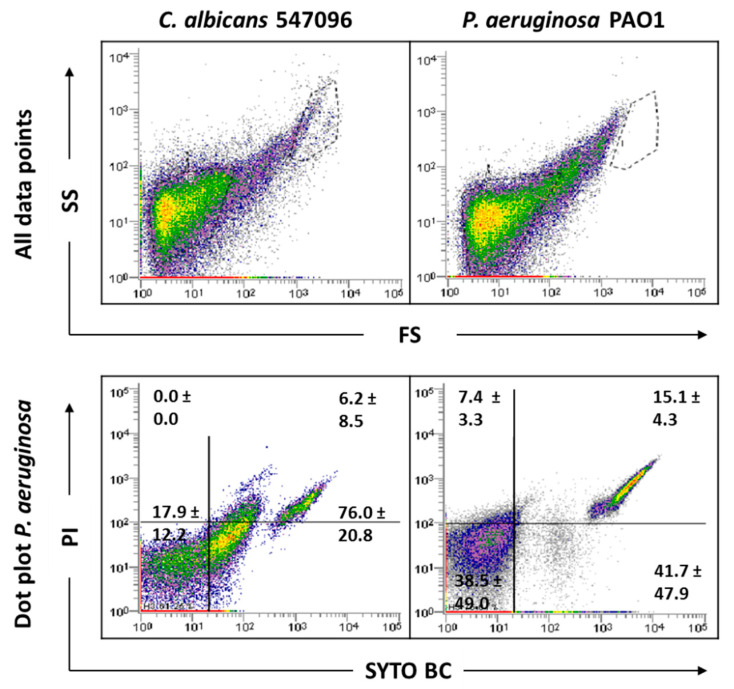
Representative dot plots obtained for biofilm matrix of *C. albicans* 547096 and *P. aeruginosa* PAO1 by FCM. In ‘all data points’, the dot plots SS (Side Scatter) × FS (Forward Scatter) are represented, as acquired in the logarithm. The dot plot of *P. aeruginosa* represents the areas delineated to represent bacteria.

To deeply understand what influenced the results obtained in the biofilm samples, the possible interference of the biofilm matrix was also evaluated. The EPS matrix is composed of large amounts of eDNA [3,24] that can be bounded by the used fluorochromes, but, due to its small size, is only detected by the cytometer when it is attached to other matrix components. Therefore, efforts were done to extract the biofilm matrix, then the biofilm cells with and without the matrix were analyzed on the cytometer. The FCM of the biofilm matrix samples revealed a similar pattern to that obtained previously for *C. albicans* biofilms (Figure 3). Although this alteration between planktonic and biofilm samples was not previously clearly detected for *P. aeruginosa* strains, the analyses of the biofilm matrix samples allowed us to notice that the EPS matrix also play a role in the bacterial biofilm cell counts (Figure 3).

The results indicate that both hyphal growth and the biofilm matrix account for altering the typical gate observed in *C. albicans* planktonic cultures. However, the presence of the EPS matrix has been shown to have greater impact on the FCM analysis. Although the *P. aeruginosa* gate does not differ between planktonic and biofilm, it was also evident that the matrix influences FCM analysis.

Overall, the data clearly highlighted that biofilm matrix extraction, before FCM analysis, is a requirement for this kind of methodology. Otherwise, it will not be possible to accurately analyze biofilm populations, due to the risk of populations not being properly differentiated, and consequently causing cell counts to be severely influenced by positively marked matrix events.

### 2.3. Influence of Sonication on Biofilm Cell Viability

**Figure 4:**
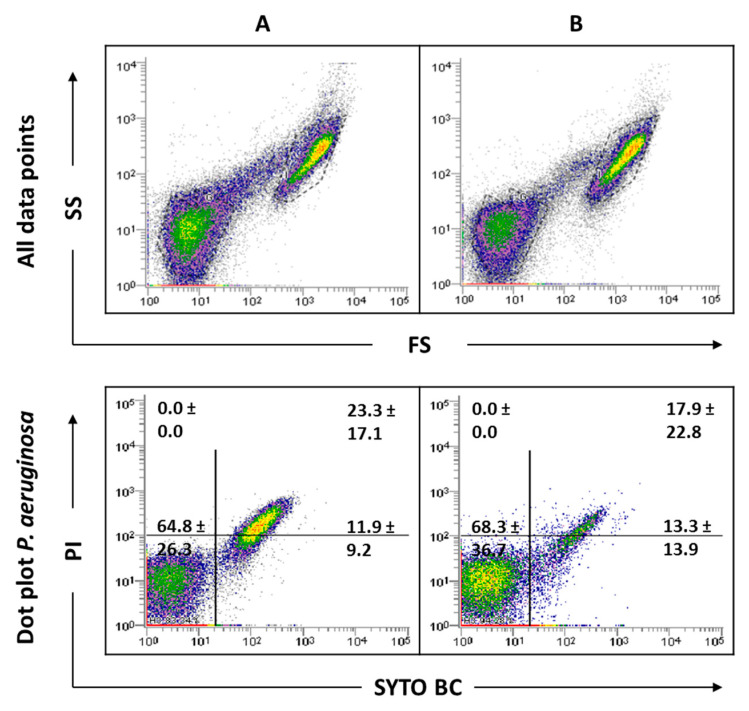
Representative dot plots obtained by FCM for *C. albicans* 324LA/94 biofilms before (**A**) and after (**B**) extraction of biofilm matrix.

As this study has mixed-species biofilms as the main focus, a sonication procedure was used based on a previous optimized protocol. The sonication time was evaluated to ensure that this procedure did not result in cell lysis (Supplementary information Figure S4). The results indicated that the matrix extraction with 30 s of sonication at 30% amplitude was not harmful to any of the strains tested (*p* < 0.05). However, these sonication conditions were not effective in removing the entire matrix of *C. albicans* SC5314 and 324LA/94 strains (Supplementary Figure S5). As can be observed in Figure 4, although some matrix has been removed from *C. albicans* 324LA/94 samples, the cellular suspensions still present some matrix traces, which are being gated where *P. aeruginosa* is usually gated, making the study of mixed-species biofilms containing this *C. albicans* strain unfeasible.

### 2.4. Analysis of Mixed-Species Biofilms

**Figure 5:**
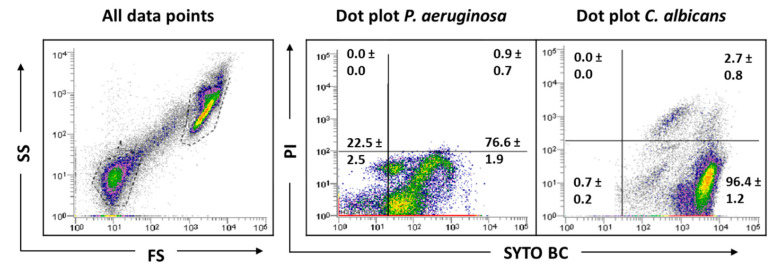
Representative dot plots obtained for *P. aeruginosa* PAO1 and *C. albicans* 547096 mixed-species biofilms by FCM. In ‘all data points’, the dot plots SS (Side Scatter) × FS (Forward Scatter) are represented, as acquired in the logarithm. Dot plots of *P. aeruginosa* and *C. albicans* represent the areas delineated to represent bacteria and fungi, respectively.

To focus and frame the study, the number of strains used in the mixed biofilm formation were narrowed to one strain for each organism. In the case of *P. aeruginosa*, as no differences were observed between the strains tested in the FCM data analysis, the PAO1 strain was selected, because it is one of the most commonly used strains for biofilm research. Regarding *C. albicans*, to minimize the effects of the biofilm matrix and to eliminate hyphae influence, 547096 was the strain selected. The sonication parameters were tested in these mixed-species biofilms, and the results show that the matrix was effectively extracted (Figure 5).

Indeed, the results showed a clear and distinct separation between bacterial and fungi populations, making the evaluation of this consortium by FCM possible. Since the *C. albicans* matrix, which was gated where *P. aeruginosa* is usually gated, does not appear because it was removed, bacterial cells will be more feasible counted.

### 2.5. Antimicrobial Effect on Mixed-Species Biofilms

**Figure 6:**
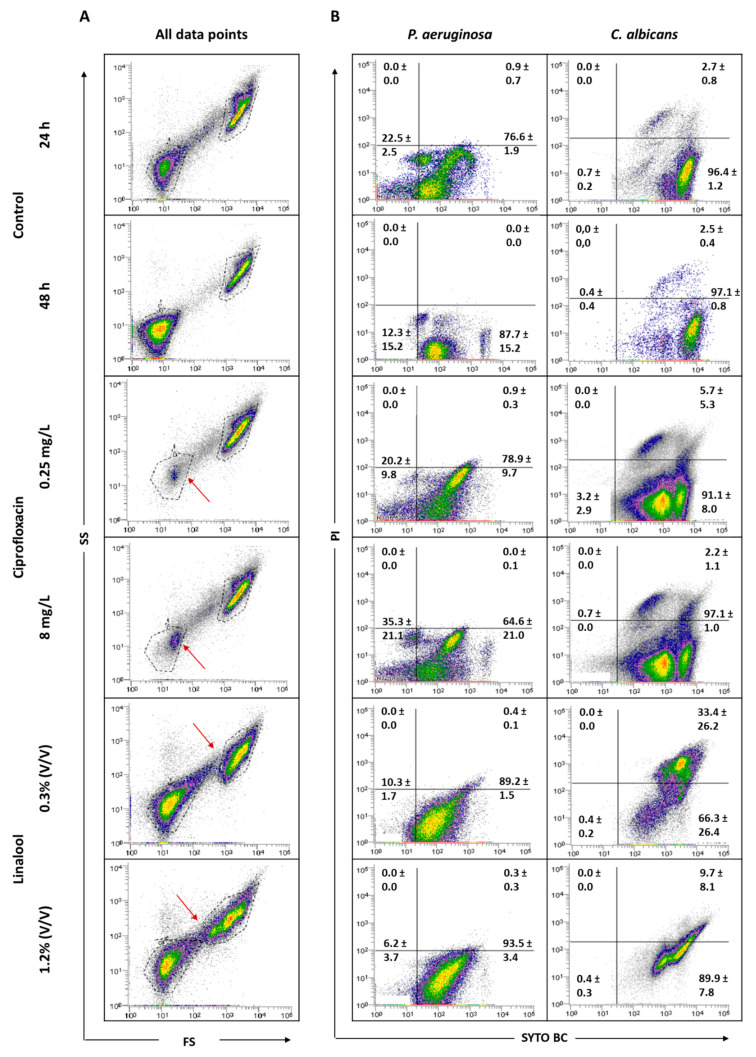
Representative all data points (**A**) and specific dot plots (**B**) obtained for *P. aeruginosa* PAO1 and *C. albicans* 547096 mixed-species biofilms by FCM. Dot plots of *P. aeruginosa* and *C. albicans* represent the areas delineated to represent bacteria and fungi, respectively. The arrows indicate alterations in the core of the population compared to the controls.

Pre-established polymicrobial biofilms were treated with ciprofloxacin or linalool and their antimicrobial effect was evaluated by FCM (Figure 6) and colony-forming units (CFU) were counted (Table 1).

To understand the effect of the antimicrobials tested, it was considered important to verify the behavior of the total population in terms of size (FS) and complexity (SS). Figure 6 displays the graphs of all data points obtained for 24 and 48 h-old biofilms, and for the populations after antimicrobial treatments. The analysis of the dot plots indicated that there are no evident changes when comparing the 24 h-old biofilms of *P. aeruginosa* and *C. albicans* with the corresponding 48 h. The core of the fungal population presented a slight increase in the SYTO BC mean fluorescence intensity (MFI) from 2189 to 4208. Regarding the effect of the antimicrobials, the data revealed that, when using both ciprofloxacin concentrations, the core of the *P. aeruginosa* population is altered compared with the controls. In terms of viability, ciprofloxacin treatment does not appear to affect *P. aeruginosa* biofilm cells. In the case of *C. albicans*, no notable differences in terms of size and complexity were observed. Nevertheless, the yeast population seemed to be slightly destabilized, with the observation of two sub-populations, as well as a decrease in the SYTO BC uptake and an increase in the propidium iodide (PI) uptake compared with the 24 h-old biofilm control. These differences were more pronounced with the lowest concentration of ciprofloxacin ranging from 2182 to 1072 for SYTO BC MFI, and from 580 to 692 for PI MFI.

Using linalool, *P. aeruginosa* and *C. albicans* populations appeared altered, being more dispersed, suggesting a more heterogeneous population. With both linalool concentrations, *C. albicans* cells presented a reduced size and complexity (Figure 6). A population displacement along the PI axis was also observed, meaning that part of the cells is double stained. Furthermore, we observed a decrease in the SYTO BC uptake compared with the control, whereas SYTO BC MFI ranged from 2189 to 1604 and 1715 with the lowest and the highest concentration, respectively. Concerning *P. aeruginosa* populations, there was no effect observed in terms of viability and only a slight decrease in SYTO BC MFI was detected (from 117 in 24 h-old biofilm to 84 after biofilm treatment).

The cellular quantifications of the untreated and treated mixed biofilms are gathered in Table 1. Overall, FCM counts were somewhat lower than those detected by CFU, except when mixed biofilms were challenged with linalool, wherein *C. albicans* CFU counts were very low or even null. However, it must be taken into consideration that the standard deviation values associated with the CFU counts are higher than those observed for FCM, which might suggest more data variability between experiments.

Regarding the FCM results (Table 1) for *P. aeruginosa*, no significant differences were observed between ciprofloxacin-treated biofilms and the 24 h-old biofilm control. Furthermore, around 2 and 2.5-log reductions were achieved with the lowest and highest concentrations of ciprofloxacin, respectively, compared to the 48 h-old untreated biofilm. Taking into consideration CFU counts, the application of ciprofloxacin gave rise to some reductions in *P. aeruginosa* cells, notably when the higher concentration was used (about 2 and 3.5-log reductions compared with 24 and 48 h-old untreated biofilm, respectively). Ciprofloxacin treatment had no relevant effect on *C. albicans* as cell numbers remain unchanged for both concentrations, whatever the method used for biofilm cell counting.

The use of linalool had no relevant effect on *P. aeruginosa* viability and culturability compared with the 24 h-old biofilms. However, when compared with 48 h-old biofilms, although not statistically significant, about a 1-log reduction was observed when using either FCM or CFU. Concerning the fungus population in the mixed consortia, the use of FCM or CFU to count biofilm cells challenged by linalool gave rise to very distinct scenarios (*p* < 0.05). Indeed, through FCM, no significant alterations in the number of cells were noticeable in comparison with the respective controls. However, CFU counts revealed a clear negative effect of linalool in *C. albicans* culturability, achieving reductions of more than 4 or 5-log compared to the 24 or 48 h-old untreated biofilms, and even total eradication with the highest linalool concentration.

## 3. Discussion

The polymicrobial nature of most infections [5] leads to the growing need for the study of these complex communities. Currently, there are several techniques that can be employed to perform biofilm analysis that are often dependent on the investigation purpose. Although FCM has been essentially applied for studying planktonic cultures [25], there are some studies using biofilms [26,27,28]. Pan et al. (2014) compared three methods (FCM, CFU and a spectrophotometry method of optical density measurement) for the quantification of bacterial cells after exposure to nanoparticles and found that FCM measurement was the quickest and most accurate method for bacterial detection [29]. This methodology has also been successfully applied in the study of single-species biofilms and to investigate bacterial physiological responses [26,27]. FCM was also successfully applied in the study of cell viability in planktonic mixed cultures, using Gram-specific fluorescent staining [30].

Since the study of polymicrobial biofilm communities using FCM is less common, the rationale behind this study was to exploit and fruitfully apply this technique in the characterization and quantification of mixed-species biofilms while paying attention to the eventual hitches that this technique could engender and trying and to find solutions to surpass them.

Biofilms are complex microbial communities in which cells are embedded in an EPS matrix that cements cells together and provides heterogeneous microenvironments, leading to cells adopting different physiological states. Thus, biofilm cells differ phenotypically from their planktonic counterparts [31]. The results obtained in this study have shown that the analysis of biofilm cell viability using FCM is not straightforward, essentially due to the presence of cells with different morphologies and the biofilm matrix. *C. albicans* biofilms are typically formed by a mixture of vegetative cells, pseudohyphae and hyphae [32], with this mixture of morphologies having been detected by FCM. Due to the size and complexity of fungal cells, it was noticed that the counts made by FCM were influenced by the presence of hyphae, since they can be counted as more than a single event (Supplementary Figure S3).

The EPS matrix represents a significant part of the biofilms, playing an important role in their development and cohesion [33,34,35]. The matrix is suggested as the biofilms’ house [36] because it protects biofilm cells from physical, chemical and biological adversities. The biofilm matrix contains several constituents, including eDNA [3,24], which, once attached to other matrix components, may be counted as positive events when passed in the flow cytometer, as highlighted by Figure 3. When the biofilm matrix was analyzed, several fluorescently labeled events, from the fungal cell gate previously defined up to the demarcated bacterial region, were detected. This squeezing of the events on the FS axis might be due to the heterogeneity of biofilm matrix components. These aspects may explain the impossibility of distinguishing the bacterial and fungal populations present in mixed cultures. Thus, matrix extraction is a crucial step before biofilm analysis by FCM. There are different methods that have been described for the extraction of EPS from single- and mixed-species biofilms, including centrifugation, filtration, heating, blending, sonication and treatments with agents or resins [37]. The EPS isolation method selected should be adapted accordingly to the type of biofilm under investigation. In the case of mixed-species biofilms, the selection and optimization of the extraction procedure was even more difficult. Moreover, it has to be taken into account that if the process becomes too complex or time consuming, the advantage of using FCM is lost. Sample sonication was the chosen methodology, but the matrix was not effectively extracted for the three *C. albicans* strains tested (Supplementary Figure S5) meaning that the success of the matrix extraction was strain dependent. These findings require longer sonication times and/or amplitudes to be tested in order to increase the amount of matrix extracted. Although it is known that sonication is an extraction method whose parameter optimization is microorganism dependent, in the case of mixed biofilms, there must be a commitment to reconcile the best possible parameters for all species present in the consortia. Thus, a reasonable time of sonication for both microbes present in the mixed cultures must be chosen, once the desirable target is achieved wherein the process does not affect the cellular viability of any of the tested species.

Based on previous studies, it can be assumed that, when using the same concentrations of antimicrobials agents, a greater antimicrobial effect occurs on planktonic cells, when comparing to the effect observed on biofilm cells, namely for ciprofloxacin [38] and linalool [39] towards *P. aeruginosa* and *C. albicans*, respectively. It is expected that, following an antimicrobial treatment, the composition and state of microbial populations might be altered [40]. Although, in some cases, there were no evident changes in the number of cells, it is important to emphasize some variations in SYTO BC and/or PI MFI, as well as variations in the core of the population (Figure 6). Ciprofloxacin is a broad-spectrum antibiotic of the fluoroquinolone class that acts on DNA gyrase (topoisomerase II) and topoisomerase IV, resulting in the inhibition of DNA replication, recombination and transcription, and thus causing bacterial death [41]. Ciprofloxacin affected the size and complexity of *P. aeruginosa* (Figure 6) and, regarding the results obtained by FCM, both concentrations of ciprofloxacin showed a bacteriostatic effect (Table 1) as the number of cells was lower after treatment compared with the 48 h-old untreated biofilm. Concerning CFU results, in addition to its bacteriostatic activity, a bactericidal activity was also observed to be more pronounced with the highest concentration of the antibiotic. These discrepancies between different methods are in accordance with the results observed by other authors [42]. Though ciprofloxacin is a bactericidal antibiotic according to classical testing, which uses planktonic cells, it is well known that cells from biofilm are more tolerant to the actions of antibiotics and harder to eradicate. Biofilm cells usually require higher doses to be eradicated by an antimicrobial [43]. The bacteriostatic effect observed here is due to the fact that cells were grown embedded in a biofilm. Both bacteriostatic and bactericidal effects of ciprofloxacin were previously reported in *Escherichia coli* [42]. Taking the 24 h-old biofilms as the reference, the application of ciprofloxacin did not alter the number of *C. albicans* cells; however, when the comparison is made with the 48 h-old untreated biofilm, a decrease in the number of cells was observed. These results indicate that ciprofloxacin might also have a fungistatic effect. Moreover, it was observed that the *C. albicans* population was divided into two sub-populations, shifted to the left with a reduced uptake of SYTO BC (Figure 6), meaning that a diminished metabolism of viable cells after ciprofloxacin treatment may have occurred. Indeed, a decrease in staining intensity was previously associated as an indicative characteristic of decreased metabolic state [26,44]. Although fluoroquinolones have no intrinsic antifungal growth-inhibitory activity, topoisomerase I and II are found in pathogenic fungi [45,46,47], which suggests that ciprofloxacin might be a strong candidate to interact with antifungal agents [48,49,50]. Linalool is a terpene alcohol commonly found as a component of the essential oils of aromatic plants. This compound has been reported as having antifungal activity, specifically against *C. albicans* [51,52]. A more recent study showed that linalool suppressed the expression of several virulence-related genes [53]. The effect of linalool on *C. albicans* cells showed marked differences between FCM and CFU counts. Although *C. albicans* did not suffer reductions in terms of FCM counts, the number of CFU was diminished after linalool application, meaning that their growth ability was affected. Even though no reduction in the FCM counts was observed, a clear alteration in population diversity, as well as a shift in the fungus population along the PI axis, was observed (Figure 6), meaning that cells are double stained with SYTO BC and PI. This increase in the number of cells being double stained suggests that linalool damages the cell membrane, allowing PI to enter into the cells. Thus, it can be assumed that part of the cell is in an intermediate physiological state between life and cell death, which may explain the reduction in the ability of fungi to grow on solid media. A double-stained population is considered injured, as discussed by Léonard et al. [54]. Therefore, the discrepancy between CFU and FCM counts may be explained by the emergence of viable but non-culturable cells; nonetheless, an additional non-culture-based method would be valuable to unambiguously assure this hypothesis. Indeed, the exposure of cell populations to antimicrobial action can lead to the appearance of different cell subpopulations, particularly an increase in the number of viable but non-culturable cells [55,56]. The differences here observed reflect the problem associated with CFU counting, since it does not allow for the detection of viable but non-culturable cells. Differences between culturable and FCM counts were previously reported by other authors [26,44]. The abovementioned linalool membrane effect was also observed in other studies, where it was reported that this antimicrobial agent acts by causing the disruption of the membrane integrity and interrupting the cell cycle [51,57]. Concerning *P. aeruginosa*, the application of both linalool concentrations did not alter the number of cells when compared to the 24 h-old biofilm control, but caused a decrease when the 48 h-old untreated biofilms were used for comparison, meaning that that linalool might have bacteriostatic activity. Similar bacteriostatic effects of linalool were previously reported for other bacteria, namely *Enterobacter cloacae*, *E. coli*, *Proteus mirabilis*, *Salmonella enteritidis*, *Salmonella typhimurium, Staphylococcus epidermidis* and *Listeria monocytogenes* [58].

In addition to the discrepancies between FCM and CFU counts already mentioned, others were also observed (Table 1). In most cases, the number of culturable cells was higher than the FCM counts. This fact can be explained by the presence of injured cells that can reverse their state on fresh culture media and thus recover their growth ability. Moreover, in general, CFU results have higher variability between experiments, which leads to less accurate results.

Overall, this study showed that FCM can be a reliable methodology for the study of mixed-species biofilms, allowing for the discrimination of the stakeholders. However, for each consortium, previous optimization procedures must be followed, namely biofilm matrix extraction methodologies. Moreover, it was demonstrated that, when FCM is applied to scrutinize the mode of action of antimicrobial treatments, new insights can be provided due to the multiparametric analysis this technique allows.

A great step forward in the present research would be the clinical implementation of this methodology. Once the protocol is well developed and it is routinely applied, it is expected that it would be applied to real biofilm communities in clinical settings using blood samples or other samples where microorganisms are founded. Although FCM has been employed much more in the field of hematology, it has already been studied in different contexts. Microbial detection by FCM has already been proven to be possible using blood samples, as demonstrated in a previous work [59]. Clinical microbiology has undergone important changes during the last few years. Indeed, in recent years, microbiological techniques used in laboratories have been increasingly complemented by cutting-edge technologies such as FCM. The use of these practices presents several advantages to others, such as culture-dependent methods or microscopic approaches, since it provides multiparametric single-cell analysis very rapidly.

## 4. Materials and Methods

### 4.1. Microorganisms and Culture Conditions

Three reference strains of *P. aeruginosa*, two non-mucoid strains (PAO1 and UCBPP-PA14 (PA14)) and a mucoid strain (ATCC 39324), were used throughout this work. In addition, two clinical isolates of *C. albicans* (324LA/94, an oral isolate obtained from the culture collection of Cardiff Dental School (Cardiff, UK) and 547096, a urinary isolate obtained from the culture collection of the Biofilm Group of the Centre of Biological Engineering (Braga, Portugal)), and a reference strain, SC5314, were tested.

Prior to each assay, *P. aeruginosa* and *C. albicans* strains were subcultured from the frozen stock preparations onto Tryptic Soy Agar (TSA) and Sabouraud Dextrose Agar (SDA) plates, respectively. TSA and SDA were prepared from Tryptic Soy Broth (TSB; Liofilchem S.r.l., Roseto, Italy) or SDB (Liofilchem) supplemented with 1.2% (*w/v*) agar (Liofilchem). The plates were then incubated aerobically at 37 °C for 18–24 h.

Pure liquid cultures (pre-inocula) of *P. aeruginosa* were grown overnight in TSB, whereas *C. albicans* was maintained in SDB. For biofilm assays, 0.22 µm of filter-sterilized RPMI 1640 medium (Gibco^®^ by Life Technologies^TM^, Grand Island, NY, USA) at pH 7.0 was used.

### 4.2. Biofilm Formation

Biofilm assays were performed as previously described [60], with some modifications. Briefly, the initial cell suspension (pre-inocula) was centrifuged (3000× *g*, 4 °C, 10 min) and the pellet resuspended in RPMI 1640 to achieve a concentration of ~1 × 10^7^ CFU per mL. Bacterial concentration was estimated using an ELISA microtiter plate reader at an optical density of 640 nm (OD_640_ nm) (Sunrise-Basic Tecan, Männedorf, Switzerland), while yeast cells were enumerated by microscopy using a Neubauer counting chamber. For mixed-species cultures, a combination of 50% of the suspended inoculum of each species was used. Cellular suspensions were further transferred to 24-well plates (Orange Scientific, Braine-l’Alleud, Belgium). Plates were then incubated aerobically for 24 h on a horizontal shaker at 120 rpm and 37 °C.

### 4.3. Hyphal Induction

In order to promote hyphal growth of *C. albicans* cells, planktonic cultures of *C. albicans* S5314 were grown overnight in RPMI supplemented with 2% (*v/v*) fetal bovine serum (Biochrom AG, Berlin, Germany) at 120 rpm and 37 °C. Cells were then analyzed by FCM.

### 4.4. Biofilm Quantification

#### 4.4.1. Determination of Culturable Cells

After biofilm formation, wells were washed twice with sterile water after discarding the planktonic fraction. Afterwards, 500 μL of phosphate-buffered saline (PBS; 10 mM potassium phosphate, 150 mM NaCl; pH 7.0) was added to each well and the biofilms were scraped. In order to ensure the reproducibility of the scraping method, the conditions were strictly followed in all experiments by using a pipette tip and scraping each well about 1 min. To remove any aggregates, biofilm suspensions were vigorously vortexed (V1-Plus Biosan, Riga, Latvia) for 30 s. The resulting biofilm suspensions were then serially diluted in sterile water and plated onto agar plates (TSA for *P. aeruginosa* and SDA for *C. albicans*) for single-species biofilms. For mixed-species biofilms, *Pseudomonas* Isolation Agar (PIA; Sigma-Aldrich, St. Louis, MO, USA) and SDA supplemented with 30 mg/L gentamycin (Sigma-Aldrich, St. Louis, MO, USA) (to suppress the growth of *P. aeruginosa*) were used for the specific isolation of *P. aeruginosa* and *C. albicans*, respectively. Agar plates were incubated aerobically at 37 °C for 24–48 h for culturable cell counting. Values of culturable sessile cells were expressed as log_10_ CFU per mL and represent the average of the triplicates for each strain. At least two independent experiments were carried out in duplicate.

#### 4.4.2. Extraction of Biofilm Matrix

For the extraction of the biofilm matrix, a previously described protocol was followed [61]. In brief, after washing and scraping the biofilm, all suspensions were sonicated for 30 s at 30% amplitude in a sonicator (Cole-Parmer 750-Watt Ultrasonic Homogenizer, Vernon Hills, IL, USA). The cells were then separated from the matrix by centrifugation at 3000× *g* for 5 min at 4 °C. The supernatant was filtered with a membrane pore size of 0.2 µm. The pellet, which corresponds to the cells of a biofilm without matrix, was resuspended in 1 mL of PBS to be analyzed further.

#### 4.4.3. Flow Cytometry Assay

Biofilm cell viability was also determined by FCM. In brief, pre-formed biofilms were washed twice, scraped in 1 mL of PBS, vortexed at maximum speed (30 s) and analyzed by cytometry. In addition, the biofilm cells (without the EPS matrix) and the EPS matrix itself (after matrix extraction procedure) were also analyzed. Lastly, 0.5–2 µM of SYTO BC (Invitrogen™, Carlsbad, CA, USA) and 15 µM of PI (Invitrogen™, Carlsbad, CA, USA) were added to the tested suspensions. Samples were incubated in the dark for 20 min, at room temperature, and were analyzed further in an EC800^TM^ flow cytometer (SANYO, Osaka, Japan). SYTO BC fluorescence was detected on the FL1 channel (PMT = 5) while PI fluorescence was detected on the FL4 channel (PMT = 3). SYTO BC absorbs at 485–487 nm and emits at 500–504 nm while PI excitation occurs at 535 nm and emission at 617 nm.

For all detected parameters, amplification was carried out using logarithmic scales. The cellular concentration was determined by acquiring the counts by the equipment. Multi-parametric analyses were performed on the scattering signals (forward scatter, FSC and side scatter, SSC), as well as on the FL1 (green fluorescence) and FL4 (red fluorescence) channels. When appropriate, a slight adjustment of the gate was made to guarantee the inclusion of the total population in the FCM analysis. For all assays, at least two independent experiments were carried out in duplicate.

### 4.5. Influence of Sonication on Biofilm Cell Viability

The biofilm suspensions obtained were pooled and sonicated for 30 s at 30% amplitude in a sonicator. A non-sonicated sample was included as a control. After this, for each sample, the values of biofilm-culturable cells were determined by CFU counting. On these samples, total cell counting was performed in duplicate for both species.

### 4.6. Antimicrobial Effect on Mixed-Species Biofilms

The antimicrobial effects of the antibiotic ciprofloxacin (Sigma-Aldrich) and the naturally occurring terpene alcohol, linalool (Sigma-Aldrich), were evaluated in mixed-species biofilms of *P. aeruginosa* PAO1 and *C. albicans* 547096. For this, 24 h-old pre-established mixed biofilms were exposed to defined concentrations of each antimicrobial: 0.3 or 1.2% *v/v* for linalool and 0.25 or 8 mg/L for ciprofloxacin. The rationale behind the use of these concentrations was based on the assumption that the lower concentrations had already been reported as inhibitory for planktonic culture; then, these concentrations were gradually boosted until we found one that had an inhibitory effect on biofilm without causing total cell eradication. Briefly, after biofilm formation, 500 μL of cell suspension was replaced by the antimicrobial solutions prepared at 2 times the desired concentration. Plates were then incubated aerobically at 37 °C for another 24 h. The results were assessed for biofilm cell culturability through CFU enumeration using selective growth media, as previously described, and by FCM. Both 24 and 48 h-old untreated biofilms were used to infer whether the antimicrobial agents demonstrated a bacteriostatic/bactericidal or fungistatic/fungicidal activity. At least two independent experiments were carried out.

### 4.7. Statistical Analysis

Data were analyzed using the Prism software package (GraphPad Software version 6.01). One-way ANOVA tests were performed, and means were compared by applying Tukey’s multiple comparison test. The statistical analyses performed were considered significant when *p* < 0.05.

## 5. Conclusions

Overall, the obtained data allow us to strengthen the belief that FCM is a versatile and accurate technique to analyze biofilms; however, it is crucial to take into account some technical aspects to avoid erroneous interpretations. FCM analysis is strain dependent and, as the biofilm matrix is variable for each of microorganism, either grown in isolation or in polymicrobial consortia, the method of extraction (regardless of the one chosen for this purpose) must be personalized for each case. The use of FCM to analyze mixed biofilms challenged by the application of an antimicrobial can provide important insights, explaining the alterations in the behavior of the microbial community and suggesting antimicrobial modes of action in biofilm populations. These outcomes strengthen FCM as a promising technique to study heterogeneous biofilms and evaluate the efficacy of therapeutic approaches.

Throughout this work, the pitfalls related to FCM analysis of inter-kingdom polymicrobial biofilms were fully addressed and efforts were made to circumvent them in order to reliably characterize complex biofilm communities, which are still poorly explored using this approach. This work highlights the fact that this technique can be fruitfully used in the understanding of antimicrobial studies as long as specific and tailored optimization is carried out.

## Figures and Tables

**Table 1:** FCM counting and colony-forming units (CFU) enumeration of pre-established mixed biofilms treated with two different concentrations of ciprofloxacin and linalool. Both 24 and 48 h-old untreated biofilms are included for comparison purposes. Log_10_ values represent means ± standard deviations (sd).

Condition	*P. aeruginosa*		*C. albicans*	
	FCM Counts/mL	CFU/mL	FCM Counts/mL	CFU/mL
24 h-old biofilm	5.69 ± 0.03	6.99 ± 0.45	6.11 ± 0,17	6.51 ± 0.55
48 h-old biofilm	7.37 ± 0.02	8.53 ± 0.48	6.87 ± 0.10	7.46 ± 0.69
Ciprofloxacin (0.25 mg/L)	5.36 ± 0.24^#^	6.08 ± 0.94 ^#^	6.44 ± 0.23	6.64 ± 0.82
Ciprofloxacin (8 mg/L)	4.85 ± 0.62^#^	4.99 ± 1.31 *^,#^	6.43 ± 0.23	6.82 ± 0.44
Linalool (0.3% *v/v*)	6.26 ± 0.21	7.62 ± 0.70	6.07 ± 0.19	2.02 ± 1.91 *^,#^
Linalool (1.2% *v/v*)	6.27 ± 0.07	7.38 ± 0.41	6.28 ± 0.03	0.00 *^,#^

* Significantly different compared with 24 h-old biofilm control (*p* < 0.05). ^#^ Significantly different compared with 48 h-old untreated biofilm (*p* < 0.05).

## Supplementary Materials

The following are available online at https://www.mdpi.com/2079-6382/9/11/741/s1. Figure S1: Representative dot plots obtained for planktonic and biofilm cells of *P. aeruginosa*, Figure S2: Representative dot plots obtained for planktonic and biofilm cells of *C. albicans*, Figure S3: Representative dot plots obtained by FCM of planktonic cells of *C. albicans* SC5314 (A) and hyphal growth induction (B), Figure S4: Effect of sonication process on biofilm cell viability. CFU enumeration of *C. albicans* (A) and *P. aeruginosa* (B) biofilms before and after 30 s of sonication at 30% amplitude, Figure S5: Representative dot plots obtained for *P. aeruginosa* and *C. albicans* biofilms (A) and biofilm cells after matrix extraction (B) by FCM.
