# Supplementary material for: Pitfalls Associated with Discriminating Mixed-Species Biofilms by Flow Cytometry
Source: Antibiotics (Basel). 2020 Oct 27;9(11):741. doi: 10.3390/antibiotics9110741 (PMC7694060; doi:10.3390/antibiotics9110741)
Supplement: Supplementary file 1 [file antibiotics-09-00741-s001.pdf]

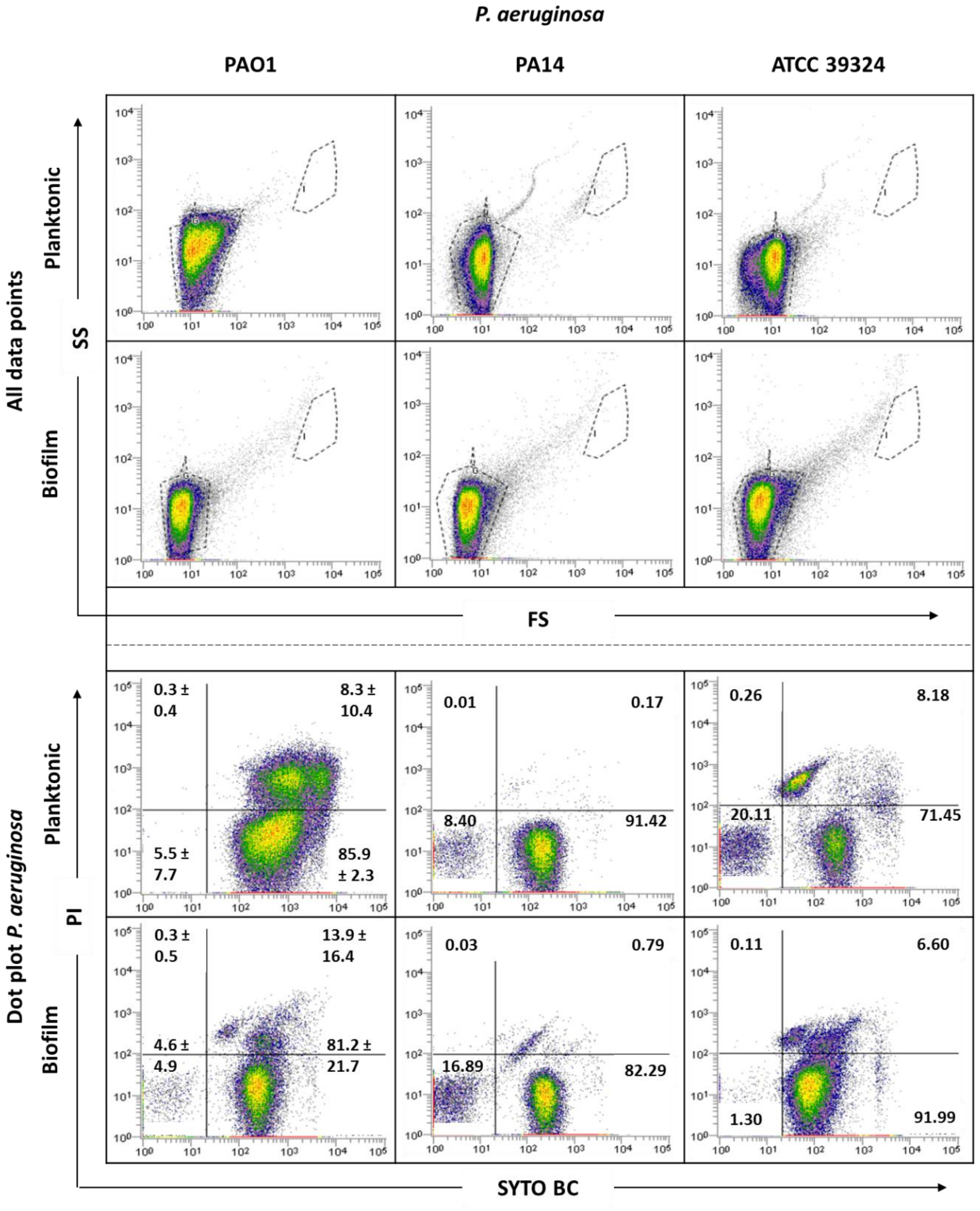

Figure S1. Representative dot plots obtained for planktonic and biofilm cells of *P. aeruginosa*.

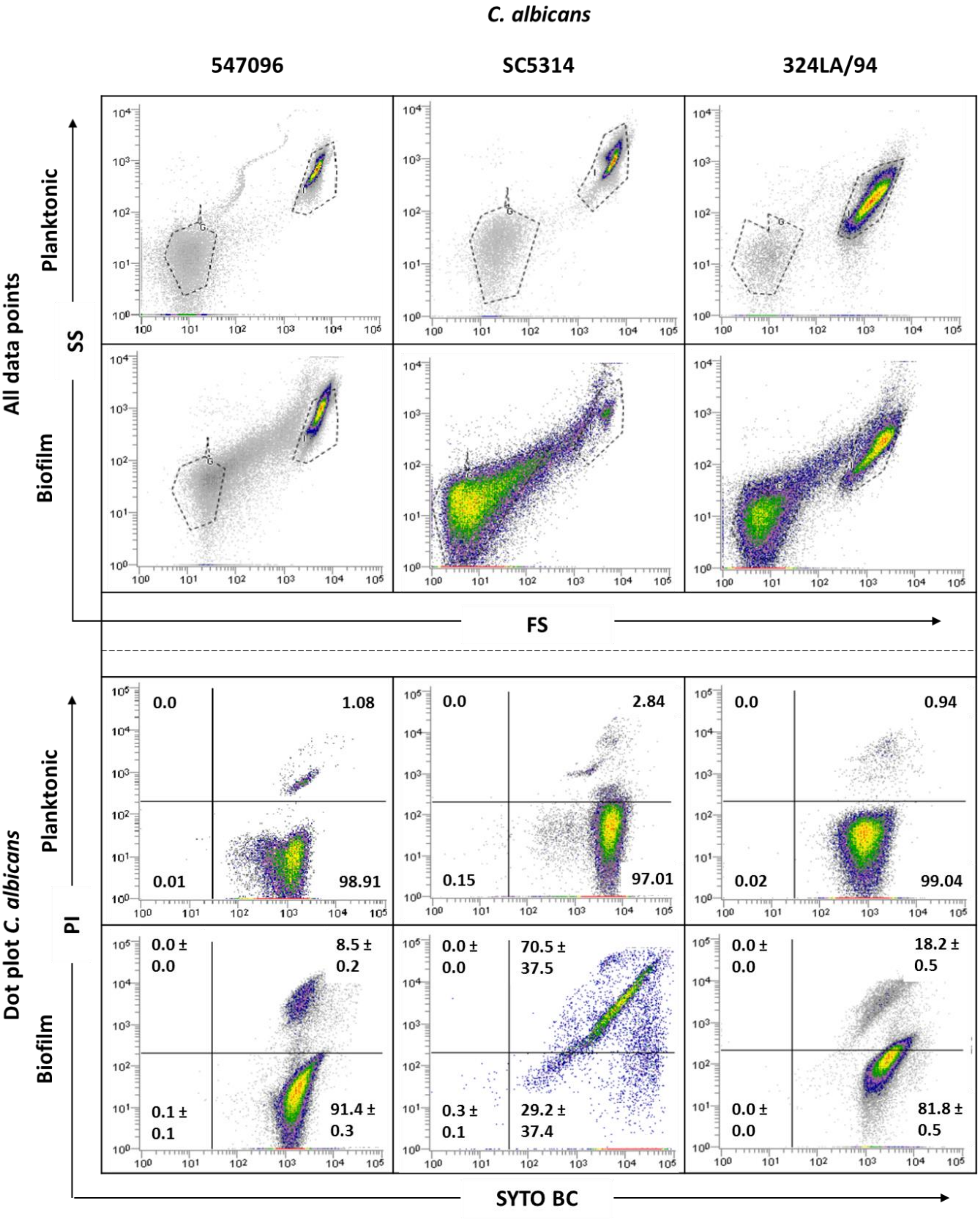

Figure S2. Representative dot plots obtained for planktonic and biofilm cells of *C. albicans.*

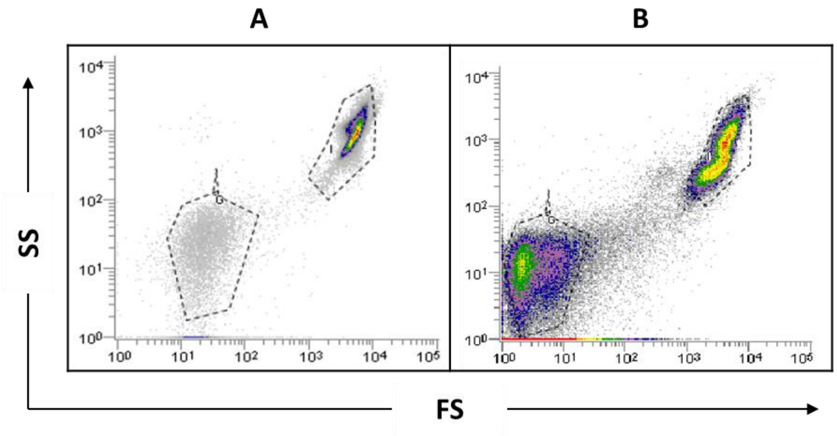

Figure S3. Representative dot plots obtained by FCM of planktonic cells of *C. albicans* SC5314 (**A**) and hyphal growth inducing (**B**).

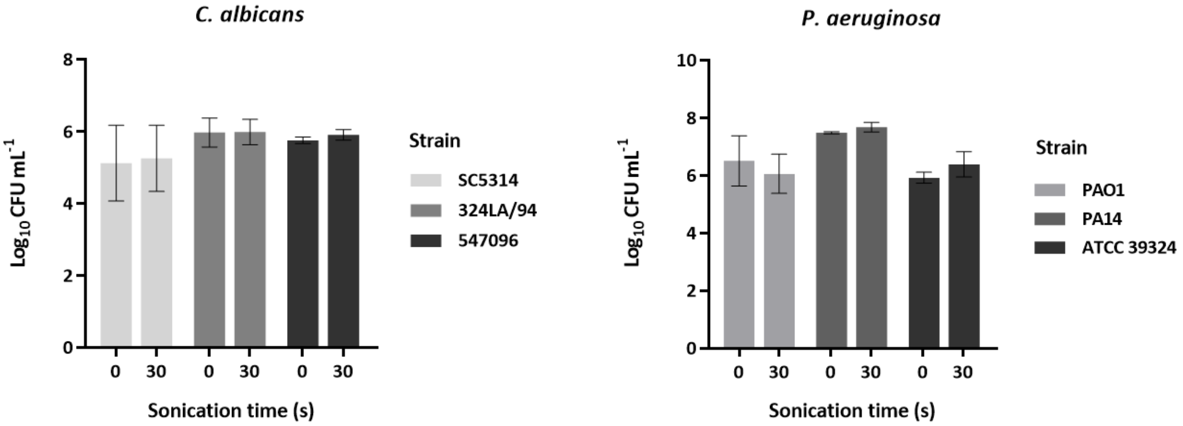

Figure S4. Effect of sonication process on biofilm-cells viability. CFU enumeration of *C. albicans* (**A**) and *P. aeruginosa* (**B**) biofilms before and after 30 s of sonication at 30 % amplitude.

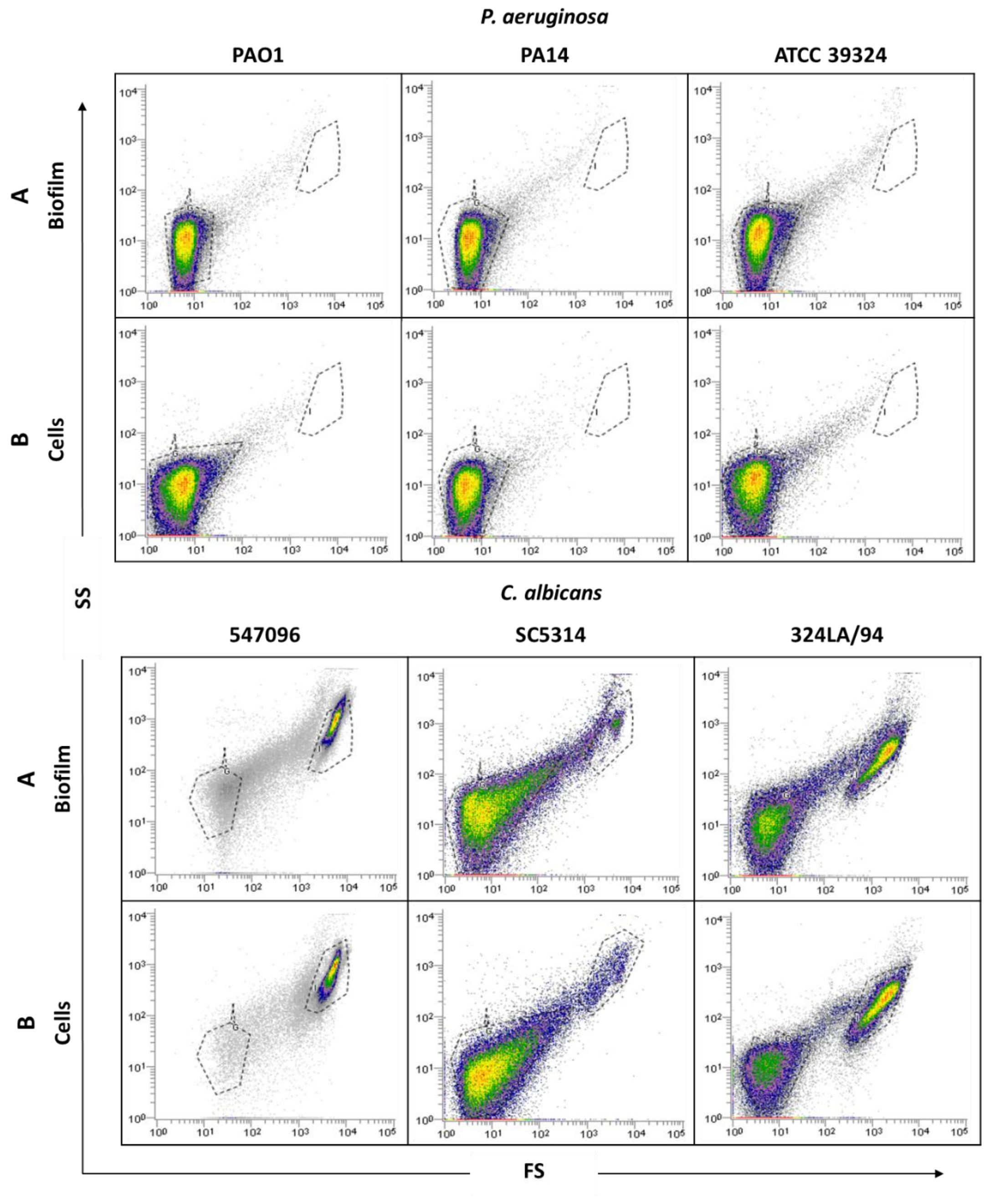

Figure S5. Representative dot plots obtained for *P. aeruginosa* and *C. albicans* biofilms (A) and biofilm cells after matrix extraction (B) by FCM.
